# 3,3′-Diindolylmethane protects cardiomyocytes from LPS-induced inflammatory response and apoptosis

**DOI:** 10.1186/s40360-018-0262-x

**Published:** 2018-11-09

**Authors:** Qiang Luo, Ankang Yang, Quan Cao, Hongjing Guan

**Affiliations:** 1 Department of Cardiology, Renmin Hospital of Wuhan University, Wuhan, Hubei 430060 People’s Republic of China; 2 Cardiovascular Research Institute, Wuhan University, Wuhan, Hubei 430060 People’s Republic of China; 3 Hubei Key Laboratory of Cardiology, Wuhan, Hubei 430060 People’s Republic of China

**Keywords:** DIM, LPS, Septic cardiomyopathy, ROS, H9C2, NFκB

## Abstract

**Background:**

3,3′-Diindolylmethane (DIM) has been extensively studied as a potential therapeutic drug with free radical scavenging, antioxidant and anti-angiogenic effects. However, whether DIM has similar effects on cardiomyocytes remains unknown. Here we evaluated DIM’s influence on inflammation and apoptosis of H9C2 cardiomyocytes induced by LPS and to explore the possible mechanism of the effects.

**Methods:**

H9C2 cells were incubated with DIM (10, 20 and 30 μM) with or without LPS for 24 h. The cytotoxicity of DIM was detected by CCK-8. The levels of tumour necrosis factor (TNF)-α and interleukin (IL)-6 were then measured using RT-qPCR and ELISA. Cell apoptosis rate and reactive oxygen species (ROS) content after DIM treatment were measured by flow cytometry. Expressions of NFκB, P-NFκB, IκBa, P-IκBa, Bax and Bcl-2 after DIM treatment were detected by western blot. The rate of NFκB nuclear translocation after DIM treatment was determined by immunocytochemical analysis.

**Results:**

LPS stimulation promoted TNF-α and IL-6 mRNA expression. After treatment with various concentrations of DIM (10, 20 and 30 μM), TNF-α and IL-6 mRNA expression was clearly impaired, especially in the LPS + DIM30(μM) group. ELISA was used to measure TNF-α and IL-6 concentrations in cellular supernatant, and the result was verified to be consistent with RT-qPCR. Additionally, DIM treatment significantly blocked LPS-induced oxidative stress and inhibited LPS-induced apoptosis in H9C2 cardiomyocytes according to the results detected by flow cytometry. Moreover, compared with LPS alone, DIM significantly inhibited the LPS-induced phosphorylation of NFκB (p-NFκB) and Bax expression and increased Bcl-2 expression.

**Conclusions:**

DIM may have a protective effect for H9C2 cardiomyocytes against LPS-induced inflammatory response and apoptosis. DIM may be a new insight into the treatment of septic cardiomyopathy.

## Background

Sepsis and septic shock are critical illnesses in intensive care unit, they have a high mortality and seriously affect the quality of life to patients [1]. In patients with severe sepsis, multi-organ dysfunction especially myocardial dysfunction commonly occurs [2]. Sepsis is the body’s response to infectious factors, the immune system can produce a series of inflammatory cascades which leads to myocardial depression and other organ dysfunctions [3]. There is a broad consensus that the standard therapy for septic myocardial depression should focus on the infection control and maintenance of normal blood pressure [4].

In sepsis-induced cardiomyopathy, the function and structure of myocardial tissue are damaged. Chemical mediators, including cytokines and endotoxins, appear to be the main pathogenic factors [4]. Inflammatory cytokines are a class of endogenous polypeptides produced by immune system cells with many powerful biological effects that mediate a variety of immune responses. High expression of inflammatory cytokines in the body can cause a variety of so-called heart failure phenotypes, including myocardial dysfunction and progressive left ventricular dysfunction in the patients with sepsis [4, 5]. TNF-α and IL-6 are two of cytokines. Previous studies have indicated that exposure to lipopolysaccharides (LPS) can effectively promote the production of TNF-a and IL-6 in H9C2 cardiomyocytes via activation of the nuclear factor (NF)-κB signalling pathway [6, 7]. Researchers have reported that TNF-α and IL-6 can contribute to myocardial inhibition [7, 8]. Concomitant inflammation and oxidative stress induced by LPS can further exaggerate myocardial cell damage [9–11]. Persistent stress of inflammation and oxidation eventually leads to cardiomyopathy, apoptosis and cardiac function damage. Thus, blocking the activation of the NFκB signalling pathway and supressing oxidative stress may be clinically effective in the patients with septic shock.

DIM is a compound extracted from the brassica plants and is a product obtained by catalytic condensation of indole-3-methanol (I3C) in the body. Studies have shown that DIM has a number of beneficial characteristics, including free radical scavenging, antioxidant and anti-angiogenic effects, and DIM can promote tumour cell apoptosis [12–14]. Cho et al. have found that DIM can ameliorate the inflammatory response by blocking NFκB cell signal transduction pathway [15]. However, whether DIM has a similar effect on sepsis-induced cardiomyopathy remains unknown. Thus, our aim was to explore the potential therapeutic effect of DIM in LPS-stimulated H9C2 cardiomyocytes and to clarify the underlying mechanism.

## Materials and methods

### Reagents and experimental drugs

Dimethyl sulfoxide (DMSO), 3,3′-Diindolylmethane(≥98%purity) and LPS were obtained from Sigma (St. Louis, MO, USA). LPS was dissolved in PBS (1 μg/ml). DIM was firstly dissolved in DMSO, and then dulbecco’s modified eagle medium (DMEM) (Hyclone, Utah, USA) cell culture medium containning 10% fetal bovine serum (FBS, Gibco Company, USA) was used to dilute DIM to the concentrations of 10, 20, and 30 μmol/L (μM). Phosphate buffer saline(PBS) were purchased from Hyclone (GE, Utah, USA). Trypsin and penicillin were procured from Beyotime (Jiangsu, China). Primary anti-bodies of P-NFκB, NFκB, P-IκBa, IκBa were obtained from Cell Signaling TechnologyInc (CST, Boston, USA). Primary anti-bodies of GAPDH, Bax and Bcl-2 were procured from Abcam (Cambridge, UK). HRP-Goat anti Rabbit of secondary antibodies were obtained from ASPEN (WuHan, China). ROS detection kit was from Beyotime. Annexin V-FITC Apoptosis Detection kit was provided by Tianjin Sungene (TianJin, China).

### Cell culture

H9C2 cells were provided by Hubei Key Laboratory of Cardiology (Wuhan, China). The cells were maintained in DMEM culture medium supplemented with 10% FBS in a humidified incubator at 37 °C with 5% CO2. H9C2 cells were digested with 0.25% trypsin, then approximately 1 × 10^6^ cells were plated in a six-well culture dishes, and were grown at 37 °C for 24 h for the experiments.

### Cell viability assay

Approximately 10 000 H9C2 cells were plated in a 96-well culture dishes, and were grown at 37 °C for 24 h. Then, culture the cells in DMEM medium without FBS for 12 h. After that, cell supernatant was discarded, then fresh medium containing 10% FBS and different concentrations of DIM (10, 20, 30 μM) with or without LPS (1 μg/ml) were used to culture the cells another 24 h. Remove the original culture, and washed the cells three times with PBS (to remove the effect of the drug on absorbance), 100 μL of PBS was added, and 20 μL cell counting kit-8 (CCK-8, Biosharp, HeFei, China) was added to each well. Put the dishes in an incubator for 3 h. According to the instructions of manufacturing company, the corresponding absorbance (A value) was measured by microplate reader (Tecan Group Ltd., Switzerland) and the values of optical density (OD) were used to assess the cell viability.

### Real-time quantitative PCR(RT-qPCR)

After DIM treatment to H9C2 cells, the mRNA levels of TNF-α and IL-6 were detected by RT-qPCR. First, the total RNA of each group was extracted by Trizol reagent, and the extracted RNA was reverse-transcribed into cDNA. The cDNA was produced from the total RNA (1 μg) using a Prime Script RT reagent kit (Takara Bio) according to the manufacturer’s protocol. RT-qPCR was performed using SYBR® Premix Ex Taq™(TaKaRa, Japan) and StepOne™ Real-Time PCR instrument. The reaction conditions were as follows: pre-denaturation at 95 °C for 15 s, annealing at 58 °C for 20 s, extension at 72 °C for 45 s, and a total of 40 cycles. GAPDH was used as an internal reference. Primers were synthesized by Wuhan Jinkairui Bioengineering Co., Ltd.(Wuhan, China). The following primers were used: IL-6, forward 5`-TGGAGTTCCGTTTCTACCTGG-3` and reverse 5`-GGTCCTTAGCCACTCCTTCTGT-3`; TNF-α, forward 5`-CACCACGCTCTTCTGTCTACTG-3` and reverse 5`-GCTACGGGCTTGTCACTCG-3`. GAPDH, forward 5`-CGCTAACATCAAATGGGGTG-3` and reverse 5`- TTGCTGACAATCTTGAGGGAG-3`. The levels of IL-6 and TNF-α mRNAs were normalized to the levels of GAPDH mRNA, and relative expression was calculated according to the 2^-∆∆Cq^ method. Three replicates were used per sample.

### Enzyme-linked immunosorbent assay(ELISA)

The cellular supernatant of each sample was collected after the treatment with DIM. The contents of IL-6 and TNF-α in the cellular supernatant were detected by ELISA kits according to manufacturer’s protocol.

### ROS

Commercial kits obtained from Beyotime were used to determine the levels of ROS in the H9C2 cells. H9C2 cells of each group were digested with 0.25% EDTA, and the cells were collected and washed three times with PBS. Next, removed the medium, 1 ml DCFH-DA (diluted to a final concentration of 10.0 μM) was added to each group, and incubate these cells at 37 °C for 25 min. Washed these cells four times with high glucose DMEM. The fluorescence intensity of each group measured by flow cytometry (Becton-Dickinson, New Jersey, USA) was the intracellular ROS intensity.

### Annexin V-FITC/PI apoptotic analysis

Cells treated with or without DIM were digested and harvested, washed four times with pre-cold PBS. Annexin V-FITC and Propidium Iodide (PE-Annexin V/7-AAD kit) was applied to stain these cells. Then, flow cytometry was used to detect the stained cells and assess the apoptosis ratio.

### Western blot

To extract total protein, the cells of each group were harvested and washed three times; these cells were dissolved in the radioimmunoprecipitation assay (RIPA) lysis buffer. Protein content was quantified using a protein concentration assay kit (ASPEN, WuHan, China). The obtained proteins sample were separated according to the molecular weight by SDS-polyacrylamide gel electrophoresis, and the protein separated on the gel was transferred to the PVDF membrane (Millipore, MA, USA) for western blot analysis. Blots were visualized with an ECL chemiluminescent detection kit (ASPEN, WuHan, China). The AlphaEaseFC software (Alpha Innotech, California, USA) was used to analyse the optical density of the target bands.

### Statistical analysis

The experimental data was given as means±SEM. SPSS 24.0 statistical software was used for statistical analysis. The results were analyzed using one-way analysis of variance. *P* < 0.05 was considered statistically significant.

## Results

### Effect of DIM on cell viability

**Fig. 1:**
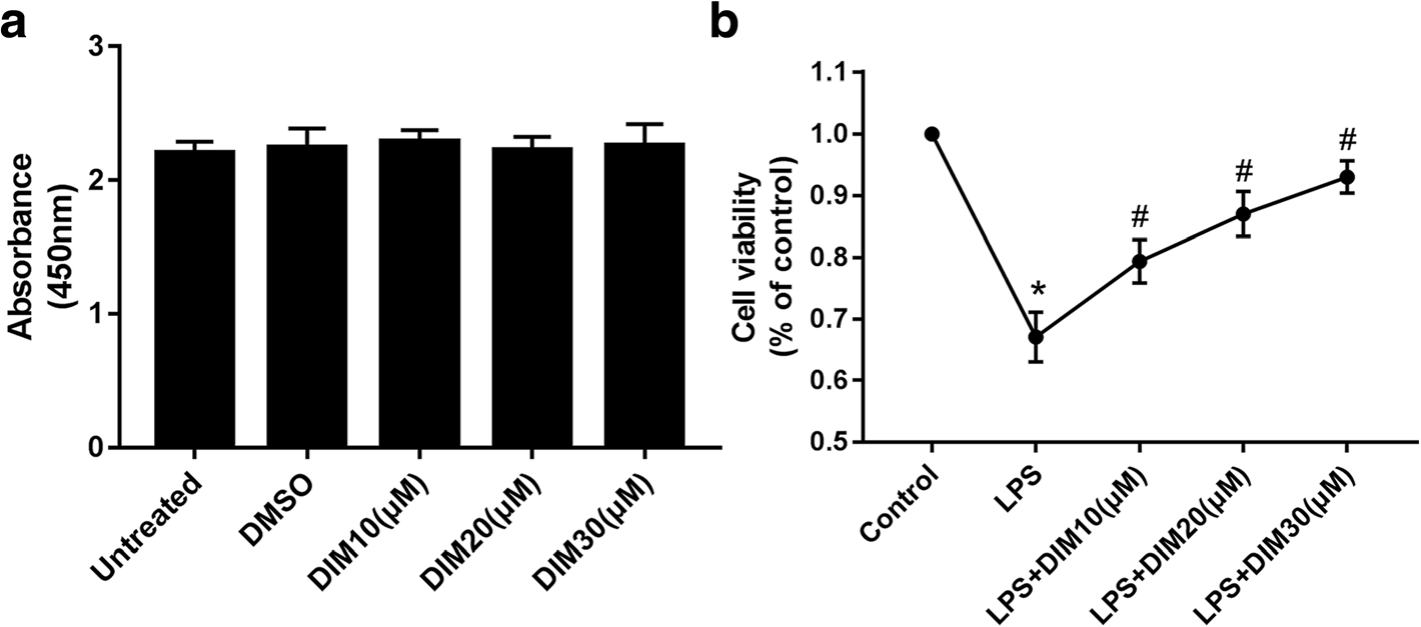
**a** Shows the effects of DIM on cell viability in H9C2 cells. **b** Shows the cell viability curves associated with LPS stimulation with or without DIM (10, 20, 30 μM) treatment of H9C2 cells. *n* = 9. * means *P* < 0.05 vs. the control group; # means P < 0.05 vs. the LPS group. Error bars indicate SEM

As shown in Fig. 1a, the results showed that the difference of cell viability between DIM-treated cells and the untreated group had no statistical significance, suggesting that DIM (10, 20, 30 μM) had no cytotoxic effect in H9C2 cells at these concentrations. Here, 0.1% DMSO was shown to have no effect on H9C2 cell viability and was used as a control group in the subsequent experiments. From Fig. 1b, we can see that LPS causes a decrease in cell viability of H9C2 cardiomyocytes by approximately 35%, but DIM treatment attenuates LPS cytotoxicity in a dose-dependent manner.

### DIM decreased the expression levels of TNF-α and IL-6

**Fig. 2:**
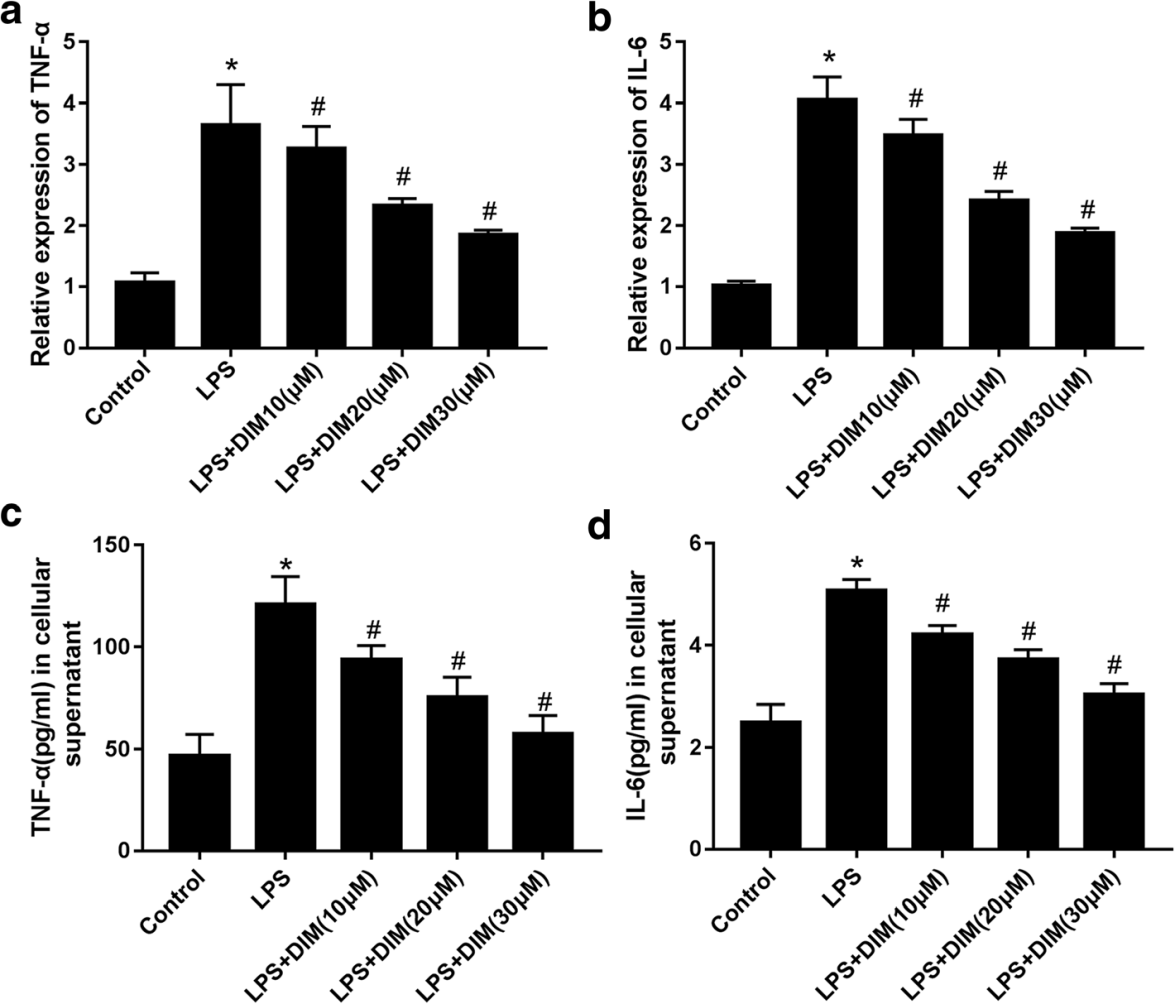
Effects of DIM on TNF-α and IL-6. The mRNA level were determined by RT-qPCR (**a**, **b**); The cellular supernatant contents were determined by ELISA. *n* = 3. * means P < 0.05 vs. the control group; # means P < 0.05 vs. the LPS group. Error bars indicate SEM

As known to all, the typical characteristics of inflammation is the elevated levels of inflammatory factors, inclouding TNF-α and IL-6, so we detected the levels of TNF-α and IL-6 by RT-qPCR and ELISA. LPS significantly upregulated mRNAs encoding TNF-α (Fig. 2a) and IL-6 (Fig. 2b) by approximately 3.6-fold and 4-fold, respectively. DIM prevented an elevated mRNA level of TNF-α and IL-6 in a dose-dependent manner.

To confirm the anti-inflammatory effect of DIM, ELISA was used to detect the levelof TNF-α and IL-6 culture supernatant. The observed levels were well consistent with the real-time quantitative PCR results (Fig. 2c, d). These data demonstrate that DIM effectively inhibited the inflammatory response induced by LPS in H9C2 cardiomyocytes.

### DIM attenuated LPS-induced oxidative stress

**Fig. 3:**
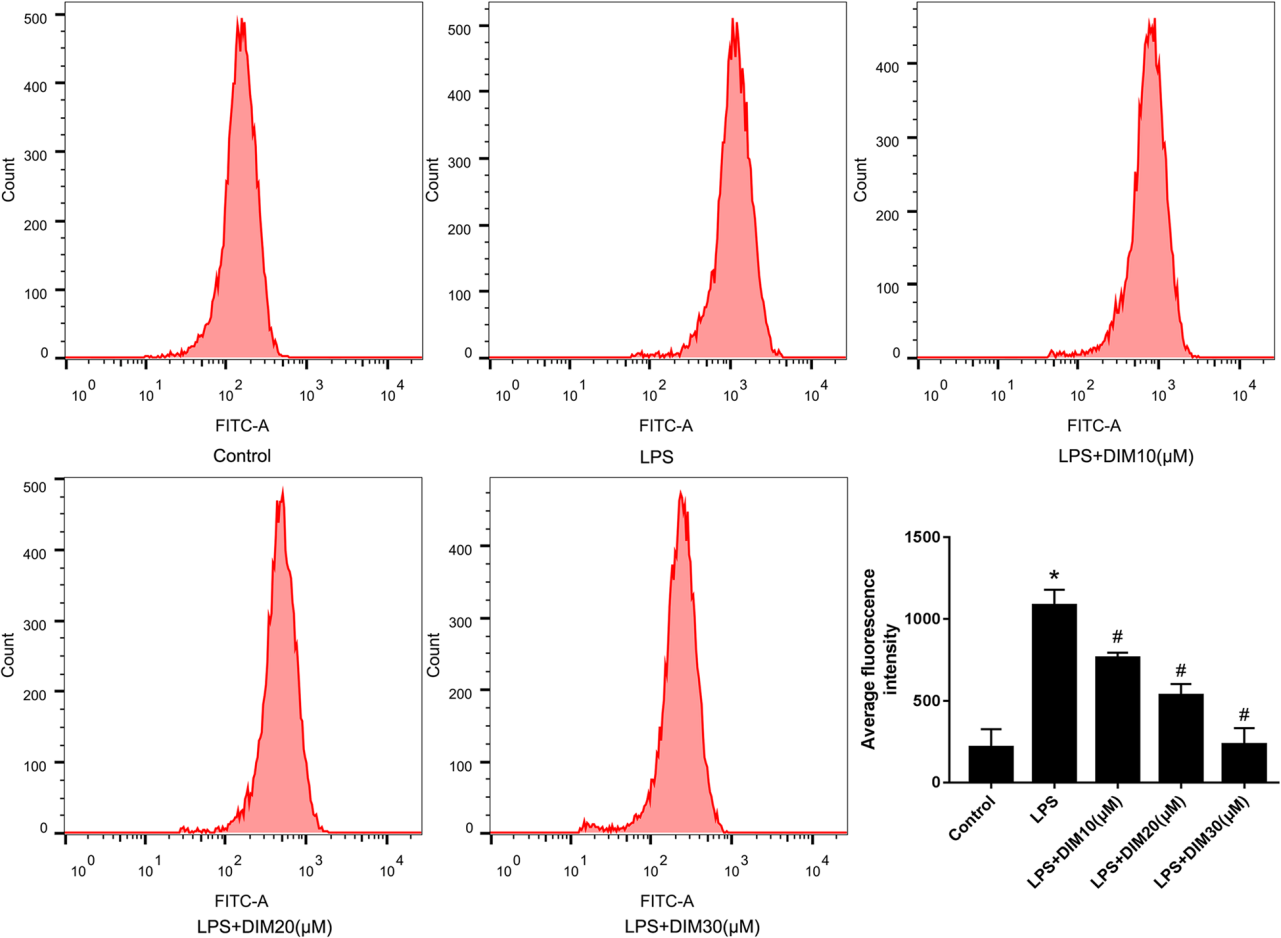
DIM decreased ROS production. The level of ROS was measured by flow cytometry. As indicated in Fig. 3: * means P < 0.05 vs. the control group; # means P < 0.05 vs. the LPS group. Error bars indicate SEM

It has been evidenced that ROS promoted cardiomyocyte apoptosis and inhibited myocardial function [16]. We detected LPS-induced ROS production after DIM treatment. To detect the concentrations of ROS, DCFDA (redox-sensitive fluoroprobe) was used and flow cytometry was used to measure the fluorescent signal. As shown in Fig. 3, after treatment with LPS for 24 h, the ROS levels in the H9C2 cells were significantly increased. However, this increase was attenuated by DIM treatment (Fig. 3). ROS are the main cause of oxidative stress, these results suggest that DIM blocks LPS-induced oxidative stress.

### DIM attenuated LPS-induced apoptosis

**Fig. 4:**
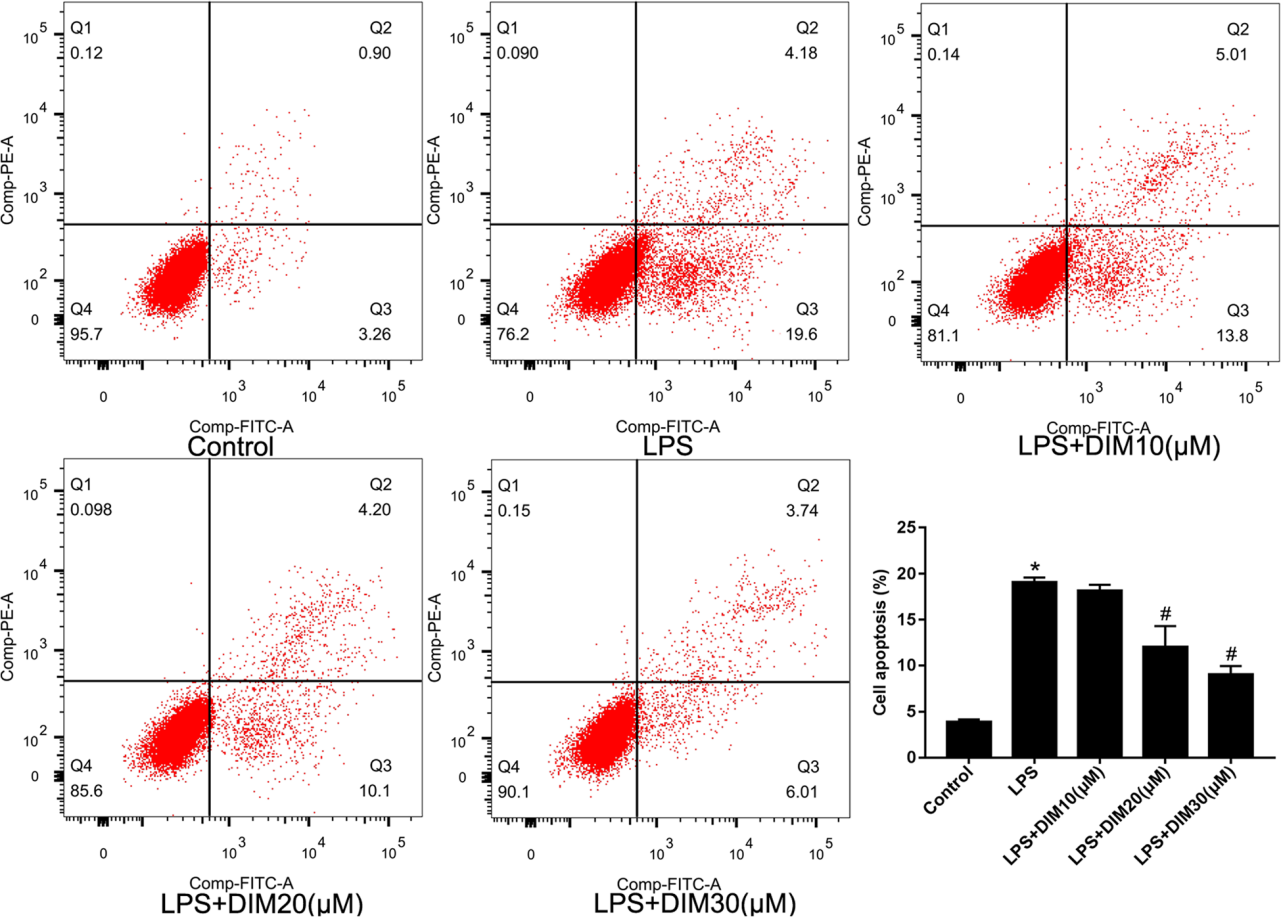
DIM inhibited LPS-induced cell apoptosis. Figure 4 is the representative apoptosis images. n = 3; * means P < 0.05 vs. the control group; # means P < 0.05 vs. the LPS group

Severe inflammation and oxidative stress can lead to cardiomyocyte apoptosis. In our study, apoptosis ratio of each group was detected by flow cytometry. Excessive proportion of cardiomyocyte apoptosis means the likelihood of myocardial dysfunction increases. As shown in Fig. 4, Cells in the lower right quadrant and upper right quadrant are defined as apoptotic cells.

As shown in Fig. 4, only 3.91 ± 0.15% of the cells were apoptotic in control group. After LPS treatment, apoptosis ratio was observed a significant increase (19.11 ± 0.28%); however, DIM treatment lowered the apoptosis ratio in H9C2 cells in a dose-dependent manner. Higher concentrations of DIM (30 μM) lowered the apoptosis ratio by 40 to 50% of that in the LPS group. (9.03 ± 0.53%) (Fig. 4).

**Fig. 5:**
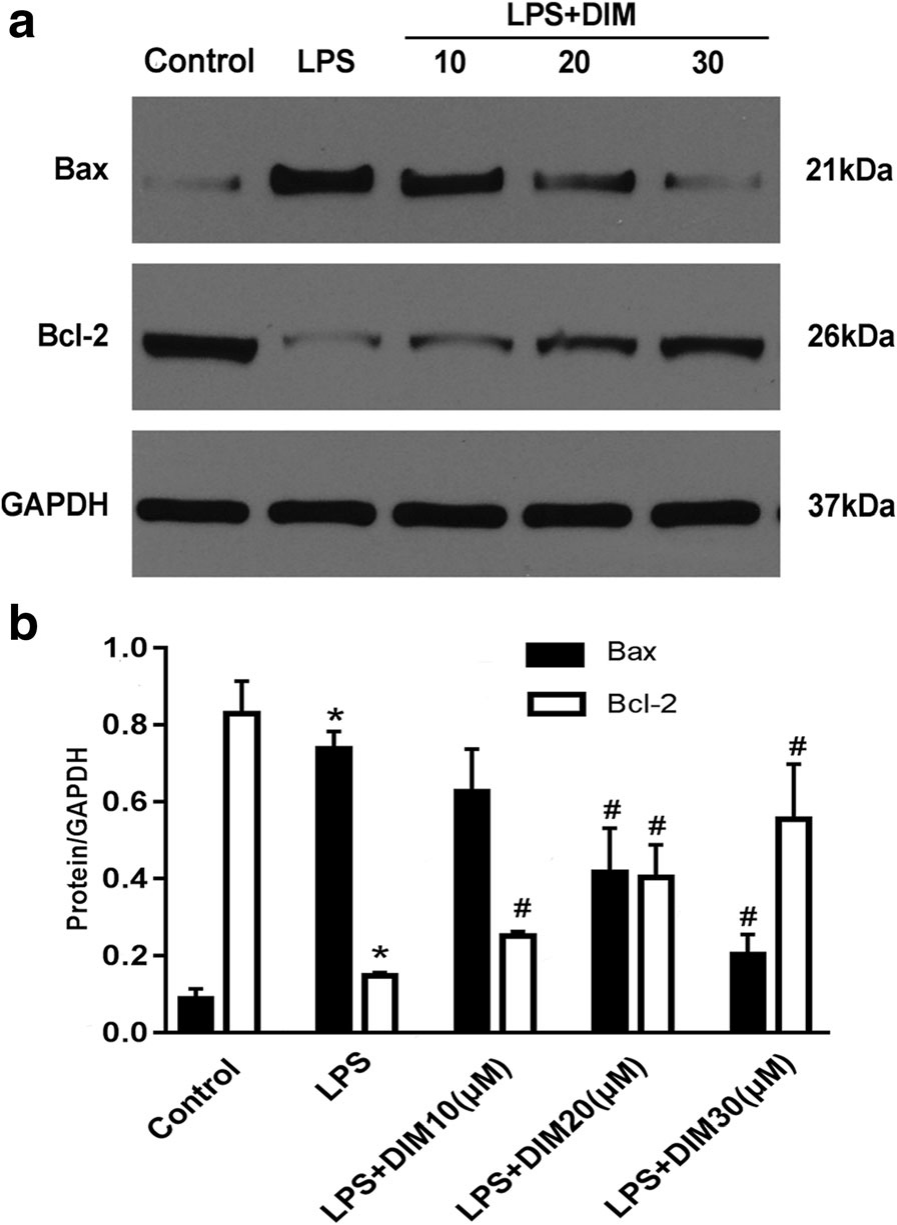
Effect of DIM on the protein level of Bax and Bcl-2. **a** Representative western blot images. **b** DIM inhibited Bax expression but promoted Bcl-2 expression after LPS. n = 3; * means P < 0.05 vs. the control group; # means P < 0.05 vs. the LPS group

Bcl-2 and Bax are involved in the administration of cell apoptosis. The Bcl-2/Bax ratio is a “molecular switch” that initiates apoptosis. Therefore, we detected the expressions of these two proteins. From Fig. 5, we can see that 24 h of LPS stimulation significantly reduced Bax expression and decreased Bcl-2 expression, whereas DIM treatment increased Bcl2 expression and decreased Bax expression, indicating that the anti-apoptotic property of DIM may be related to the regulation of Bax and Bcl-2 expression.

### DIM regulated the NFκB signalling pathway

**Fig. 6:**
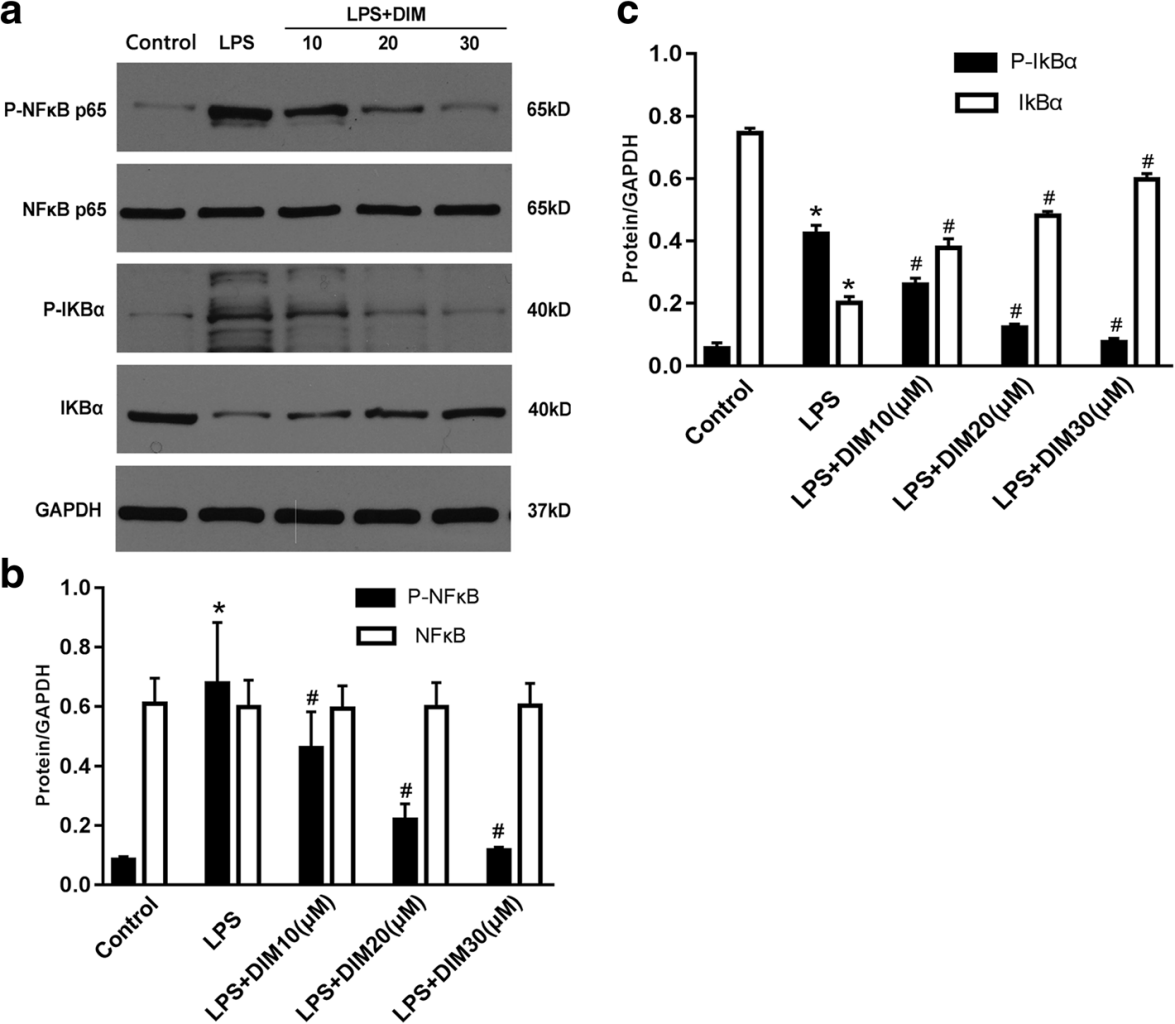
Effect of DIM on NFκB signal pathway cascade (**a**, **b** and **c**). DIM decreased the phosphorylation levels of NFκB. **a** Representative western blot images. **b** Quantitative results of NFκB and p-NFκB. **c** Quantitative results of IκBa and P-IκBa. n = 3; * means P < 0.05 vs. the control group; ^#^ means *P* < 0.05 vs. the LPS group

We then explored in molecular detail the mechanisms underlying the anti-inflammatory and anti-apoptotic properties of DIM. In cardiomyocytes, the NFκB signalling pathway has been shown to be associated with the expression of inflammatory factors, including IL-6 and TNF-α [17]. IκBα phosphorylation via IKKβ results in I-κBα degradation and NFκB release. NFκB translocates into the cell nucleus, thereby leading to the expression of its downstream target molecules. In our study, western blot was conducted to determine the phosphorylation levels of p65 and IκBα. LPS treatment significantly promoted the phosphorylation of NFκB and IκBα. However, DIM treatment significantly blocked the LPS-induced NF-κB and IκBα phosphorylation, and subsequently decreased the degradation of IκBα (Fig. 6b, c).

**Fig. 7:**
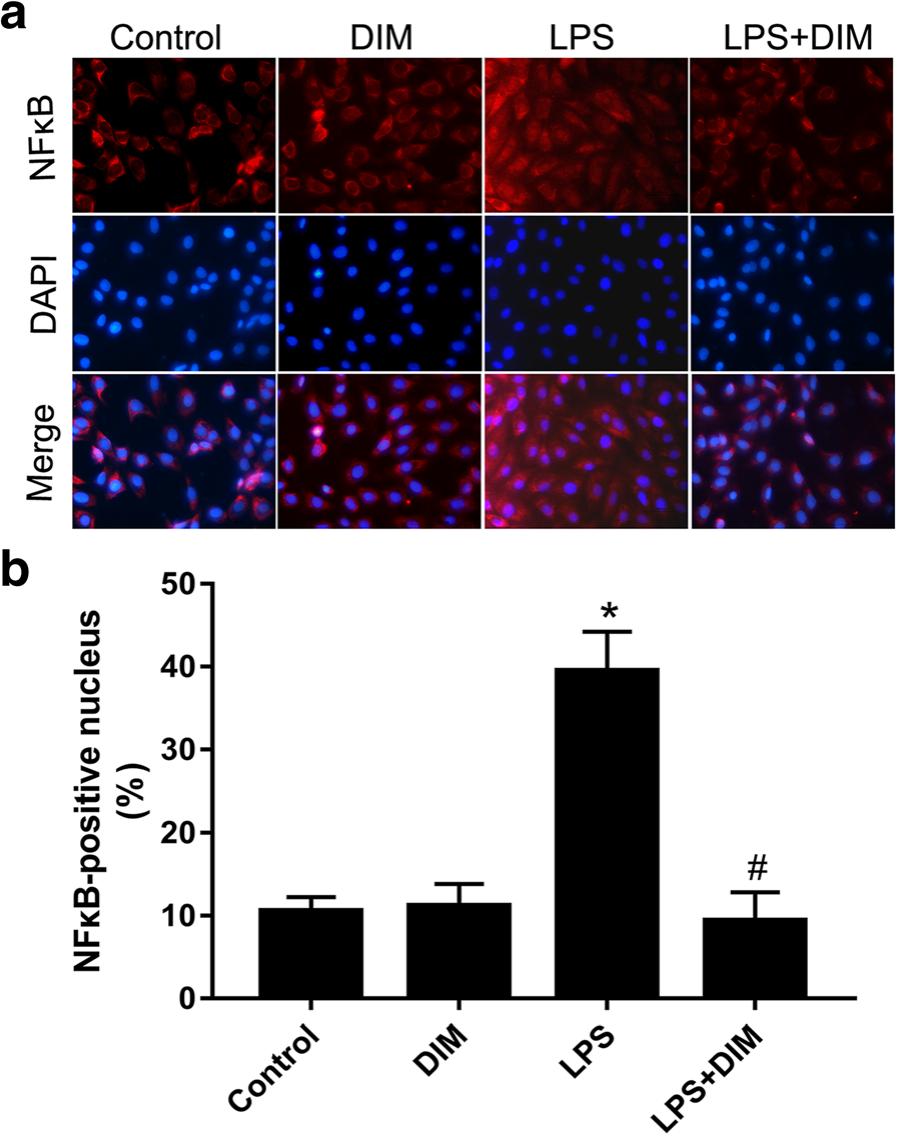
DIM inhibited NFκB nuclear translocation induced by LPS (magnification, × 400). n = 3;* means P < 0.05 vs. the control group; # means P < 0.05 vs. the LPS group

NFκB translocation was determined by immunocytochemical analysis. DIM blocked the nuclear translocation of NFκB in response to LPS. (Fig. 7b).

## Discussion

Our study has demonstrated that DIM inhibited LPS-induced inflammatory response. Moreover, DIM was an effective antioxidant, as evidenced by a decrease in ROS after DIM treatment. These properties of DIM induced a decrease in apoptosis of H9C2 cells and may be related to the suppression of the NFκB pathway. These results suggest that DIM may be able to protect the H9C2 cells from inflammation and apoptosis via the NFκB signalling cascade and may suppress oxidative stress.

Inflammation caused by infection has been suggested to be closely related to sepsis-induced myocardial depression [18]. LPS is a thick (8-10 nm) lipopolysaccharide substance located at the outermost layer of gram-negative bacteria and it involves in the initiation of plenty of pathophysiological cascades [19]. Therefore, LPS is a typical molecule used in the sepsis models in in vitro investigations. Cardiomyocytes stimulated by LPS can release large amounts of TNF-α and IL-6 [4, 5]. And a growing body of evidence demonstrated their important roles in the sepsis-induced cardiomyopathy. TNF-α can induce a negative inotropic effect in the heart resulting in decreased blood pressure and cardiac pumping function [20]. Moreover, in a patient suffering from sepsis, cardiac function gets a short-term improvement after the use of an anti-TNF antibody indicating that TNF may be associated with sepsis [21]. Pathan et al. [8] demonstrated that IL-6 was also involved in the septic shock; concentrations of IL-6 in the serum strongly predicted the extent of myocardial dysfunction in children with meningococcal septic shock. Our experiments in the H9C2 cells indicated that DIM treatment dramatically inhibited the IL-6 and TNF-α expression induced by LPS, indicating that DIM may be able to protect myocytes from inflammatory injury.

Cytokines are a group of functional proteins secreted by cells induced by internal and external stimuli and participate in the activation of transcription factors, signal transduction pathways, etc. The inflammatory cytokine cascade exerts a enormous influence on the occurrence and development of heart failure. Studies have shown that cytokines may promote the occurrence of heart failure by inducing apoptosis of cardiomyocytes. Cardiomyocyte apoptosis is a key cause of many cardiac diseases, including heart failure, atherosclerosis and sepsis-induced cardiac insufficiency [22]. Cardiomyocytes are the functional cells of the heart; their apoptosis can lead to myocardial contractile dysfunction and cardiac output reduction [23]. Numerous studies have shown that TNF-α and IL-6 can promote apoptosis [24, 25]. In TNF-α-overexpressing mice, oxidative stress and apoptosis in the cardiomyocytes were significantly more severe than those in the control group [26, 27]. Additionally, TNF-α overexpression can reduce the expression of Bcl-2 and promote cardiomyocyte apoptosis [28], eventually contributing to an disequilibrium between Bcl-2 and Bax. It has been demonstrated that the disequilibrium between Bcl-2 and Bax determines whether the cells are going into apoptosis [29]. In this study, we demonstrated that DIM can increase the level of Bcl-2 and inhibit Bax expression, and this result is consistent with the inhibitory effect of DIM on apoptosis of cardiomyocytes as evidenced by flow cytometry.

Although the entire mechanism of the protective effects of DIM on the cardiomyocytes remains unclear, according to our investigation, the regulation of the NFκB signalling pathway may be involved in these beneficial effects. NFκB is typically present in the cytosol as a preformed trimer complex. When NFκB is not activated, it binds to the inhibitory protein IκBa to form the IκBa/NFκB complex. After stimulation by LPS, IκBa is phosphorylated and eventually ubiquitinated and degraded. The NFκB dimer can be transferred into the cell nucleus and can initiate transcription and translation of various target genes including the inflammatory factors [30–32]. Tomar S et al. [33] showed that DIM treatment attenuates the LPS-mediated acute liver failure by suppressing Toll-like receptor signaling. Kim HW et al. [34] demonstrated that DIM suppresses brain inflammation by attenuating inflammatory transcription factor NFκB. In agreement with these studies, treatment with DIM inhibited the LPS-stimulated phosphorylation of NFκB and IκBα and subsequently decreased the degradation of IκBα. In our IF analysis, DIM significantly decreased the NFκB nuclear translocation indicating a decline in the NFκB activation. These results suggest that DIM inhibits the activation of the NFκB signalling pathway and attenuates inflammation induced by LPS.

Studies have confirmed a vital role of ROS in cardiovascular complications in sepsis [9, 10]. Oxidative stress factors, such as Sod2, GPX1, etc., can efficiently suppress apoptosis of the myocardial cells and prevent the LPS-induced cardiac insufficiency and death [9, 10]. In our study, we have found an exciting evidence suggesting that DIM may decrease the ROS levels. Additionally, Yao et al. [35] demonstrated that DIM protected the mouse cardiac fibroblasts from the H2O2-induced oxidative stress and decreased the generation of ROS in the adriamycin-stimulated cardiac fibroblasts. Our findings suggest that DIM has an antioxidant effect. However, we do not know the exact mechanism of the antioxidant stress function of DIM. Subsequent studies will focus of the details of the antioxidant stress function of DIM and how it can be precisely targeted by DIM.

## Conclusion

Thus, DIM may protect H9C2 cardiomyocytes from LPS-induced inflammatory response and apoptosis via inhibiting the synthesis of inflammatory factors and attenuating oxidative stress. Elucidating the molecular basis of the protective characteristics of DIM may be beneficial for developing therapeutic drugs to septic cardiomyopathy.

## Abbreviations

*CCK-8*: Cell Counting Kit-8
DIM: 3,3′-Diindolylmethane
*DMSO*: Dimethyl sulfoxide
ELISA: Enzyme-linked immunosorbent assay
IL: Interleukin
LPS: Lipopolysaccharides
NFκB: Nuclear factor-κB
*PBS*: Phosphate-buffered saline
ROS: Reactive oxygen species
*RT-qPCR*: Reverse transcription-quantitative polymerase chain reaction
*SEM*: Standard error of mean
TNF: Tumor necrosis factor
